# Perception and awareness of the public about presbyopia and its corrective approaches in Saudi Arabia: a population-based survey

**DOI:** 10.1186/s12889-024-19508-4

**Published:** 2024-07-20

**Authors:** Ali M. Alsaqr, Abdulrahman M. Alasmi, Raied Fagehi, Abusharha Ali

**Affiliations:** Department of Optometry, College of Applied Medical Sciences, King Saud University, Riyadh, Saudi Arabia

**Keywords:** Presbyopia, Near vision, Reading spectacles, Visual acuity, Emmetropia

## Abstract

**Background:**

This study investigated patients’ awareness of presbyopia and its management approaches and their preferred methods for near vision correction.

**Methods:**

In Saudi Arabia, 785 participants (aged between 35 and 60 years) completed a structured survey online, consisting of hard copies and direct interviews. The survey consisted of twenty-eight items divided into three parts. It was designed to record participants’ awareness of and preferences for presbyopia and its refractive corrections. Nonparametric tests and descriptive analyses were conducted to analyse participants’ responses.

**Result:**

Approximately half of the participants had difficulty with near vision activities, such as reading newspapers or using mobile phones. Among all the participants, 76% were not aware of presbyopia. The prevalence of uncorrected presbyopia was 48% of the 785. The majority (82%) felt that spectacles were acceptable for correction of presbyopia. Most reported that they did not experience social stigma when using reading spectacles (87% of participants). When asked if they were aware of management approaches other than spectacles, 72% responded with not at all. Most participants had no earlier knowledge of the use of multifocal contact lenses or eye drops for presbyopia correction (67% and 82%, respectively). In the present study, some tendencies to use corrective approaches to presbyopia other than spectacles were noted. Finally, participants’ age, sex, region, education, and income had a statistically significant impact on essential parts of their responses (*p* < 0.05).

**Conclusion:**

Presbyopia is a highly prevalent age-related ocular disorder, and a significant percentage of cases are uncorrected due to a lack of awareness or reluctance to wear spectacles. More efficient health education about presbyopia and its corrective alternatives is urgently needed.

## Introduction

Presbyopia can be defined as the progressive loss of eye’s ability to accommodate a focus on nearby objects [1]. It is an age-related public health concern that impairs near vision and can be characterized by a gradual decrease by the age of 40 years [2, 3]. It is believed that this age-related change is a result of a gradual loss of viscoelasticity of the intraocular lens [3].

The World Report on Vision in 2019 concluded that the global need for eye-care services is predicted to increase significantly in the future, posing a substantial challenge to health systems; this would also indicate the need for managing presbyopia [4]. In 2015, the prevalence of presbyopia was reported to be approximately 25% of the global population, among which approximately 45% of people are living with uncorrected presbyopia [5]. Specifically, it has been estimated that the number of correctable vision impairments due to uncorrected presbyopia worldwide is approximately 1.8 billion individuals [5]. Among those, 826 million have no or inadequate vision correction [5]. This prevalence is anticipated to increase with the growing ageing population [6]. Uncorrected presbyopia would result in a global economic burden, estimated to be approximately US$25 billion, due to productivity losses [7–9]. Specifically, approximately 90% of the burden of vision impairment due to presbyopia comes from low- and middle-income countries, where the estimated presbyopic correction rate is as low as 10% [3, 9, 10]. In particular, the use of digital devices for work and leisure has exponentially increased in recent decades [11]. Individuals may experience additional stress resulting from uncorrected and sometimes under corrected presbyopia [12]. Additionally, as presbyopia involves challenges in performing simple tasks such as using a smartphone or reading a newspaper, it has been found to negatively impact individuals’ daily activities, emotional well-being and quality of life [8, 13–15]. It has also been reported that presbyopia correction with spectacles increases the productivity of workers compared with uncorrected workers, especially those in jobs that require intensive near vision performance [8]. Furthermore, it has been suggested that some risk factors, such as sex (higher in women), increased sunlight exposure, an occupation that requires significant hours of near vision work, and the level of hyperopia, could impact the early onset of presbyopia [16].

### Presbyopia management

McDonald et al. suggested that presbyopia can be classified into mild presbyopia, requiring + 1.25 dioptres (D) of added power, moderate presbyopia, requiring between >  + 1.25 D and + 2.00 D, and advanced presbyopia, requiring >  + 2.00 D of added power [17]. Presbyopia can be managed with various highly effective procedures that may involve spectacles, contact lenses (CL), surgical correction and eye drops [1, 8]. Spectacle correction can be reading, bifocal, or multifocal, and the reading pairs can be bought as ready-made or can be obtained from the optometry clinic. One of the limitations of multifocal spectacles is impaired depth perception, which may lead to an increased risk of falls [18, 19]. Correction of presbyopia with CL can assume many forms; for example, mono-vision CL corrects one eye for distance vision while correcting the other eye for near vision [20]. The main limitations of this approach are reduced vision at the intermediate distance and reduced depth perception. Therefore, multifocal CL could be a better functional correction for all distances [20]. However, it has been previously reported that only one participant (out of 17) continued wearing multifocal lenses daily after six months of follow-up [21]. Furthermore, surgical treatment for presbyopia has taken many forms in recent decades. However, surgical intervention is irreversible, and each type of surgical intervention has challenges and risks that cannot be overlooked, including but not limited to the risk of infection, reduced contrast sensitivity and optical aberrations [22–26].

Recently, it was reported that presbyopic correction using pilocarpine drops could help increase the performance of near vision without negatively influencing distance vision [27, 28], which could encourage many patients living with uncorrected presbyopia to use correction and improve their quality of life. This possibility is especially attractive for patients who fear social stigma while using reading spectacles or for patients who do not like to wear spectacles every time near vision is needed. However, there are side effects of pilocarpine, including headaches, inferior night driving, dimness, ocular surface symptoms and dizziness, and a more serious increased risk of retinal detachment and iritis [29–31]. Finally, new approaches are in the final stage of development or within clinical trials that are directed toward different structures of the eye, including the cornea, sclera, lenticular lens, pupil, or other structures [32].

Presbyopia affects individuals differently depending on multiple factors but, most importantly, unaided distance vision and refraction. For example, in myopic patients, especially those with low myopia (< -3.00 dioptres), living with uncorrected near vision is usually sufficient because they can remove distance-correcting spectacles and see clearly. For more myopic patients, reading spectacles may be needed depending on the scenario and their visual demand. The challenge arises from the idea of clear near vision at a single focal point, which is strongly dependent on the degree of myopia. The proposed solution to this problem is the use of multifocal near vision spectacles, which were found to improve near vision and quality of life in both low- and high-myopic patients [33].

In Saudi Arabia, presbyopia correction may depend mainly on ready-made reading glasses (+ 1.00 to + 3.00 in 0.50D steps), which are available from major retail stores and pharmacies. Other management options, including full refraction and CL, require an appointment with an optometrist.

The increased need for presbyopia awareness could, therefore, be a significant factor in managing this condition. To the best of our knowledge, there have been no prior reports of presbyopia awareness in Saudi Arabia, which halts the process of deciding the needs of any intervention regarding awareness campaigns or increasing the availability and access to corrective options. The objectives of this cross-sectional study were to investigate (pre)presbyopic patients’ knowledge and attitudes towards presbyopia and the methods in which it could be managed. Patients’ current knowledge of presbyopia was explored, including their current and preferred future methods of management, and the factors that influence these preferences were established.

The outcomes of the current study provide a better understanding of the awareness of presbyopia in Saudi Arabia and a chance to compare these results with those of previously reported studies from various regions worldwide. Additionally, action is needed to increase public awareness and offer corrective options for affected patients.

## Materials and methods

### Study design

This was a cross-sectional study. The participants were recruited between January 2022 and June 2022. The inclusion criteria were people aged ≥ 35 years who were currently living in any region of Saudi Arabia, and there were no other exclusion criteria. Although participants 35 year of age are not truly presbyopic, they could be pre-presbyopic due to uncorrected hyperope with a reduced amplitude of accommodation, and they should be prepared for presbyopia correction and aware of it.

### Sample size

To calculate the sample size required in this study, we used Epi Info, version 7 (Centers for Disease Control, Atlanta, USA). In the last 2020 population survey, the number of residents aged ≥ 35 years in Saudi Arabia was approximately 14.7 million (General Authority for Statistics, Saudi Arabia. Retrieved January 2022, https://www.stats.gov.sa/en/43). The sample size was calculated with a confidence interval of 95%, based on previous finding showing an expected frequency set at 33% [34], a design effect of 2, and a number of clusters set at 5 (northern, eastern, central, western and southern regions). The total sample size was calculated to be 680 participants.

### The survey

The survey contained twenty-eight items and aimed to assess the knowledge of the participants concerning the condition and the different management as well as the new managements (eye drops) strategies available. Most of the items were close-ended, and few questions were open-ended to allow for further elaboration.

The first part of the survey focused on demographic information. This information included age, sex, occupation, income, education, and place of residence, which were further classified into five clusters (northern region, eastern, central, western, and southern regions). In addition, the participants were asked if they had any systemic or ocular health conditions.

The second part of the survey was designed to explore awareness of presbyopia, such as if the subject had heard about the disorder. Additionally, we asked how often the participants visited an optometrist, if they have noticed any difficulty in near vision tasks such as reading or using a mobile phone, and any history of prior refractive surgery. Additionally, in this section, participants were asked if they were using any type of vision correction for distance or near vision, from where they received the prescription, and their acceptance of this management choice. The last item concerned whether the participant faced any social stigma while using reading spectacles.

The third and final part of the survey inquired about currently available management of presbyopia (spectacles, CL and surgical procedures), specifically if the participants had prior knowledge of those different managements. In this section, the participants were also asked if they were willing to use CL. This part also explored a new topical treatment for presbyopia consisting of an eye drop (pilocarpine HCI ophthalmic solution 1.25%), which was recently approved by the FDA. We asked about prior knowledge of this management, its side effects, and, finally, whether the participants preferred this type of management over spectacles or CL despite daily administration and their reason for choosing it. The last item of the survey examined the participants preferred method of management for presbyopia.

### Distribution, data collection and analysis

This survey (Arabic copy) was created using Google forms. It was distributed through emails, colleagues in other regions, and multiple social media platforms (Twitter, WhatsApp, Telegram). Paper handouts were also distributed and collected at the same events to allow more participants to enrol in the study.

The Statistical Package for the Social Sciences (SPSS) (IBM Corp., NY, USA) was used for data analyses. Nonparametric tests and descriptive analyses were conducted to explore the data. An ordinal regression test (logit model for ordinal response) was conducted to understand the influence of the participants’ age, sex, region, education and income on their answers. A *p* value < 0.05 was considered statistically significant.

## Results

### Participants’ profile

A total of 785 participants were enrolled in this study. The respondents’ demographics are listed in Table 1. Furthermore, the respondents’ background characteristics are recorded in Table 2. In terms of respondents’ occupation status, the most common answers were jobs in the education field, administrative jobs and retired/not working. The majority of the study participants were not specialized in optometry/ophthalmology-related fields (752 respondents, 95%).

**Table 1:** Demographic profile of the respondents

*Variable*	***n*, %**
*Age group*	
***35–39 years***	281 (36%)
***40–45 years***	156 (20%)
***46–50 years***	96 (12%)
***51–55 years***	124 (16%)
***56–60 years***	60 (7.5%)
***More than 60 years***	68 (8.5%)
*Sex*	*n*, %
***Men***	321 (41%)
***Women***	464 (59%)
*Region*	*n*, %
***Central Region***	498 (63%)
***Eastern Region***	53 (7%)
***Southern Region***	94 (12%)
***Western Region***	117 (15%)
***Northern Region***	23 (3%)
*Education*	*n*, %
***University***	442 (56%)
***Secondary***	121 (15.5%)
***Master's degree***	110 (14%)
***PhD***	57 (7%)
***Intermediate***	25 (3%)
***Other***	17 (2%)
***Primary***	12 (1.4%)
***Left school before any official tests/at the age of 12 years or less***	1 (0.1%)
*Income*	*n*, %
***10,000–15,000 SAR***	222 (28%)
***5000–10,000 SAR***	182 (23%)
***15,000–20,000 SAR***	146 (19%)
***Less than 5000 SAR***	139 (18%)
***20,000–30,000 SAR***	65 (8%)
***More than 30,000 SAR***	31 (4%)

**Table 2:** Cross-tabulation of the responses to “What is your preference for near vision correction?” with sex as a factor

	***Men***	***Women***	***Total***
*Spectacles*	142	141	283
*Eye drops*	76	163	239
*Refractive surgery*	58	114	172
*Contact lenses*	8	16	24
*Will not use any of them*	36	29	65
*I do not need near vision correction*	1	1	2
*Total*	321	464	785

A total of 248 (31%) respondents reported their general health history. The most frequently reported issues were diabetes (40%), hypertension (30%), cholesterol (9%) and thyroid diseases (6%). In terms of ocular health, 491 respondents (62.5%) did not report any diseases, whereas 294 (37.5%) respondents reported poor vision, dry eyes (7%), and cataracts (3%). When they were asked about previous refractive surgery, 651 (83%) respondents did not undergo any refractive surgery.

Regarding periodic visits to an eye clinic, approximately 226 (34%) respondents indicated they visited regularly. When asked about the last clinic visit was, the most common answer was 3 months (280 respondents, 36%). Lastly, 270 (34.4%) respondents used distance spectacles, and 336 (43%) respondents used reading spectacles.

### Presbyopia

Regarding presbyopia, 407 (52%) respondents reported difficulty with near vision activities, such as reading newspapers or using mobile phones. However, most of them had not heard or read about the term "presbyopia" (595 respondents (76%)). Uncorrected presbyopia was found in 48% of respondents. For those who use spectacles, the source of the prescription was collected from 409 respondents. A total of 147 (36%) respondents obtained their spectacles from optical shops, 141 (35%) respondents from private hospitals, 83 (20%) respondents from government hospitals, 35 (9%) respondents bought them off the shelf, and 3 (0.7%) respondents obtained them from primary health care centres. The vast majority of the respondents reported accepting spectacles as management for presbyopia (642 (82%) respondents) and did not face any social stigma from using reading spectacles (680 (87%) respondents).

### Respondents’ perspective on presbyopia management

**Fig. 1:**
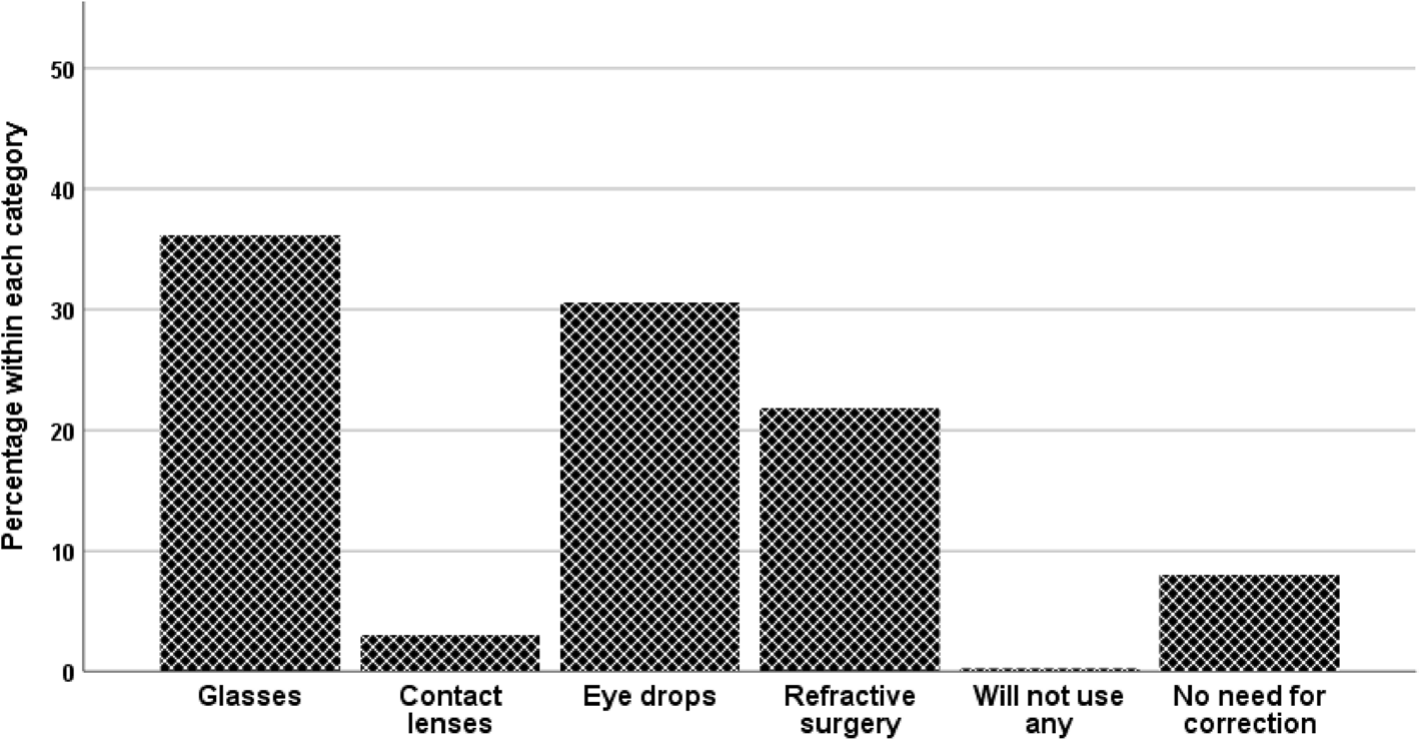
Management preferences of the study respondents

The respondents were asked about their knowledge and attitudes towards other management methods for presbyopia. A total of 565 (72%) respondents were not aware of management approaches other than spectacles. Furthermore, 524 (67%) respondents did not report any knowledge about using CL for presbyopia management. When their preferred contact lens was explored, 495 (63%) respondents rejected CL as their choice of management. The respondents were asked about their use of eye drops to manage presbyopia, but none of them used pilocarpine eye drops, as they are not yet approved by the Saudi FDA; a total of 642 (82%) respondents never heard of such management, and 694 (88%) respondents did not know how using eye drops would improve their near vision. Interestingly, 521 (66%) respondents preferred the use of eye drops, once they became available, as an alternative management for presbyopia in comparison to spectacles and CL. The overwhelming majority of respondents had no knowledge of the side effects of pilocarpine eye drops (731 [93%] respondents). The most common reasons for accepting eye drops as an alternative to other management methods were ease of use, cost, and other social reasons. Furthermore, respondents were asked about their preference for near vision correction among spectacles, eye drops, refractive surgery, CL and “will not use any correction”. The respondents’ preferential choices were diverse and are illustrated in Fig. 1. The highlighted finding was that the respondents generally preferred spectacles, eye drops and refractive surgery over CL. Finally, approximately 10% of the respondents felt they did not require near vision correction or would not use any correction.

### Impact of respondents’ demographics

An ordinal regression test (logit model for ordinal response) was performed to understand the impact of the respondents’ age, sex, region, education, and income on their responses.

#### Age

Age was found to impact only the participants’ response to the item inquiring about whether they used spectacles; the older the respondent was, the greater was the likelihood of experiencing difficulty in reading and having reading spectacles (χ2(4) = 5.12, B = 0.29, SE = 0.13, *p* = 0.024, χ2(4) = 14.1, B = 0.49, SE = 0.12, *p* < 0.0001, respectively). Additionally, age was a statistically significant determinant of the participants’ preference for near vision correction (χ2(5) = 11.9, *p* = 0.03). Younger participant were more open to the use of eye drops (when they became available) as an alternative correction for presbyopia (B = 0.52, SE = 0.26, Wald = 4.2, *p* = 0.04). Specifically, 51% of 35–39-year-olds preferred the use of eye drops as a treatment option for presbyopia.

#### Gender

**Fig. 2:**
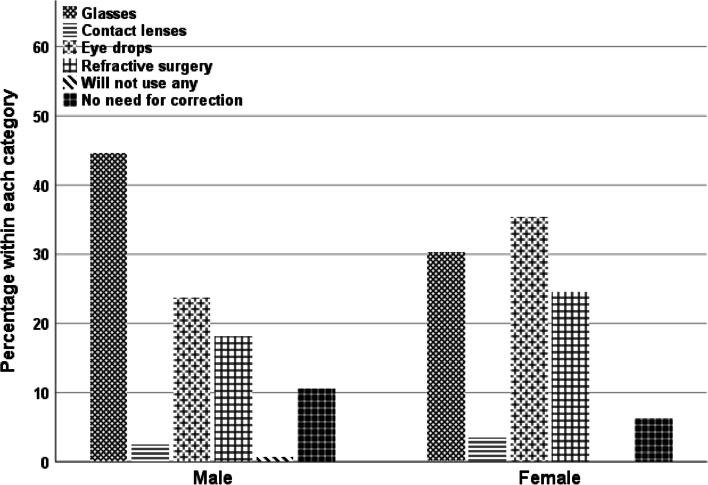
Respondents’ preferences for different managements among men and women

The ordinal regression test revealed that sex was the main determinant of all participants’ responses. First, women were relatively more aware of the term "presbyopia" than were men (χ2(1) = 5.5, B = 0.4, SE = 0.17, *p* = 0.0194), with 25% of women compared with 20% of the men being aware of the term. Women were more likely to know about using multifocal CL for near vision correction than men (χ2(1) = 9.4, B = 0.48, SE = 0.16, *p* = 0.0024), with 38% of women compared with 27% having this knowledge. The respondents’ preferences for using CL also differed between sexes (χ2(1) = 31, B = 0.86, SE = 0.16, *p* < 0.00014), with 45% of women compared with 25% of the men preferring the use of CL to correct near vision. Additionally, women were more likely to favour the use of eye drops as a management procedure (χ2(1) = 29, B = 0.79, SE = 0.15, *p* < 0.0001), as expressed by 60% of women compared with 40% of the men. Furthermore, women were more likely to use eye drops as a management procedure, as 75% responded that they would use eye drops if given as an alternative option to spectacles and CL, in contrast to 52% of men (χ2(1) = 37.6, B = 0.94, SE = 0.16, *p* < 0.00014). Finally, the respondents’ preference for near vision correction differed between sexes (χ2(5) = 31.3, B = 0.29, SE = 0.13, *p* < 0.00014) (Fig. 2). The common selection for men respondents was spectacles (B = 0.93, SE = 0.29, Wald = 10.4, *p* = 0.001), whereas for women respondents, spectacles were less favourable, and eye drops were selected more often over spectacles and other management options (B = 0.84, SE = 0.3, Wald = 7.75, *p* = 0.005) (Fig. 2).

#### Region

The respondents in different regions also significantly differed in their responses to whether they used reading spectacles ((χ2(3) = 9.34, *p* = 0.025, central region (B = 0.66, SE = 0.1, Wald = 44.99, *p* < 0.0001), eastern region (B = 0.97, SE = 0.1, Wald = 90, *p* < 0.0001), southern region (B = 1.64, SE = 0.1, Wald = 204, *p* < 0.0001) and northern region (B = 3.6, SE = 0.22, Wald = 263, *p* < 0.0001)). Specifically, reading spectacles were used by 40% of the respondents in the central region, 55% of the respondents in the eastern region, 38% of the respondents in the southern region, 54% of the respondents in the western region and 30% of the respondents in the northern region. Responses to the question about “from where they received their prescription” also differed by region or residence (χ2(4) = 10.16, *p* = 0.03, optical shop (B = -2.85, SE = 1.13, Wald = 6.32, *p* = 0.01), private medical complex (B = -2.63, SE = 1.13, Wald = 5.42, *p* = 0.02), government hospital (B = -3.15, SE = 1.15, Wald = 7.57, *p* = 0.006) and ready-made glasses (B = -2.64, SE = 1.17, Wald = 5.11, *p* = 0.02)) (Table 3).

**Table 3:** Distribution of participants’ responses to the source of reading prescription based on residence location

		Optical shop	Private medical complex	Government hospital	Ready-made glasses	Primary Health Care Centre	Total
Central Region	Count	90	80	58	20	0	248
	% within region	36.3%	32.3%	23.4%	8.1%	0.0%	100.0%
Eastern Region	Count	15	13	3	3	0	34
	% within region	44.1%	38.2%	8.8%	8.8%	0.0%	100.0%
Southern Region	Count	16	15	8	4	1	44
	% within region	36.4%	34.1%	18.2%	9.1%	2.3%	100.0%
Western Region	Count	22	31	13	7	1	74
	% within region	29.7%	41.9%	17.6%	9.5%	1.4%	100.0%
Northern Region	Count	4	2	1	1	1	9
	% within region	44.4%	22.2%	11.1%	11.1%	11.1%	100.0%

#### Education

**Fig. 3:**
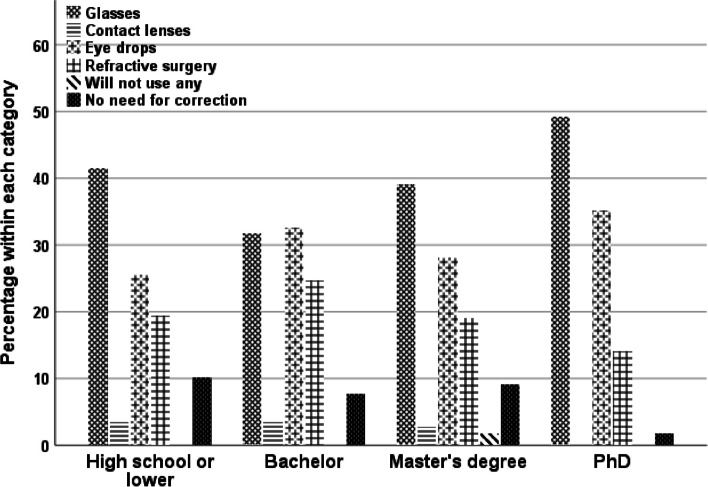
Preference for different management practices in accordance with respondents’ education. * PhD, philosophy degree

Level of education had a statistically significant impact on participants’ responses (Tables 4 and 5). In general, fewer education certificates were associated with greater difficulty in reading, least awareness of the term “presbyopia” and greater use of reading spectacles. Knowledge about the use of CL was also associated with a higher education. Additionally, the respondents’ knowledge of eye drops and their side effects was relatively low, even among those with higher education levels (Table 4). Finally, the respondents’ preference for near vision correction also varied on the basis of their level of education (χ2(5) = 14.18, *p* = 0.01), although the common choices were spectacles and eye drops across the four education groups (Fig. 3). The figure also shows that the lower the respondents’ education was, the more likely they thought there was no need for near vision correction.

**Table 4:** Ordinal regression test of variations in participants’ responses regarding education

*Variable*	*B.Sc* *442*	*MSc* *110*	*PhD* *57*	*Education of high school or less 176*	*ordinal regression test*
*Do you face a difficulty when reading from mobile or paper documents?*	53% (235)	33.5% (37)	42% (24)	63% (111)	χ2(2) = 21.95, *p* < 0.001
*Do you use reading spectacles?*	41% (183)	27% (30)	44% (25)	55% (98)	χ2(2) = 23.5, *p* < 0.001
*Have you ever heard of the term "presbyopia"?*	21% (95)	35% (39)	23% (40)	18.75% (33)	χ2(2) = 9.2, *p* = 0.01
*Did you know about using multifocal contact lenses for near vision?*	36% (159)	37% (41)	46% (26)	20% (35)	χ2(2) = 21.14, *p* < 0.001
*Do you have information about how eye drops for presbyopia work?*	8% (37)	14% (15)	17.50% (10)	26% (27)	χ2(2) = 9.13, *p* = 0.01
*Do you have information about the potential side effects of these eye drops?*	5% (22)	12% (13)	7% (4)	8.5% (15)	χ2(2) = 1.8, *p* = 0.40

**Table 5:** Crosstabulation: What is your preference for near vision correction?

Preference	B.Sc	MSc	PhD	High school or less	Total
Spectacles	139	43	28	73	283
Eye drops	144	31	20	44	239
Refractive surgery	109	21	8	34	172
Contact lenses	34	12	1	18	65
Will not use any of them	15	3	0	6	24
No need for near vision correction	1	0	0	1	2
Total	442	110	57	176	785

#### Income

**Fig. 4:**
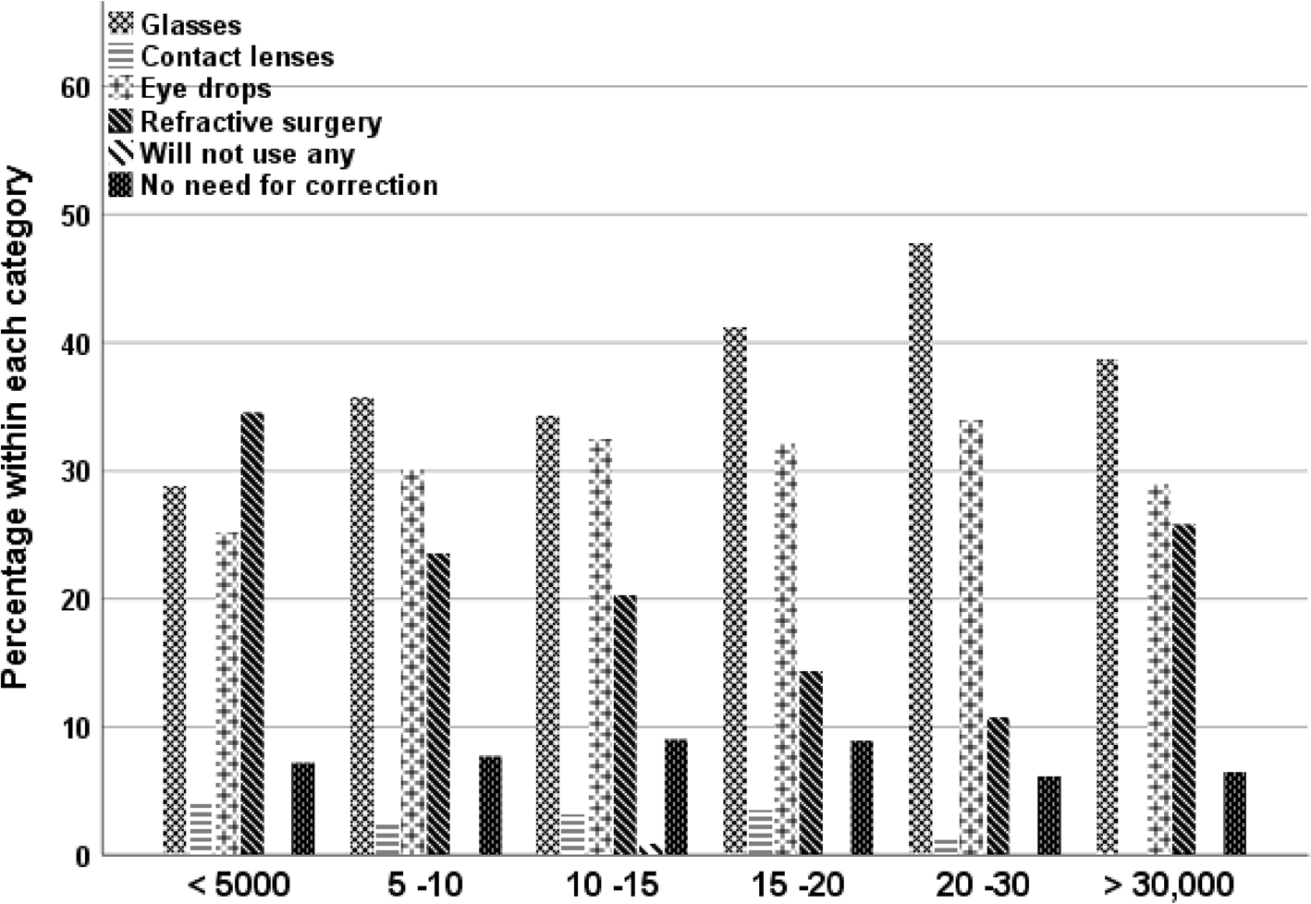
Preference of various management practices in accordance with respondents’ income. The income is listed in thousand Saudi Riyal

It has been hypothesized that income would be a determinant of participants’ preferences for presbyopia correction, as certain choices are more expensive than others. Therefore, respondents’ income was found to be significant in two responses: their preference to use CL (χ2(4) = 17.88, B = -0.11, SE = 0.13, Wald = 0.7, *p* = 0.001) and their knowledge of eye drops (χ2(4) = 12.58, B = -0.11, SE = 0.16, Wald = 0.45, *p* = 0.014). Interestingly, 35% to 45% of the respondents whose income was less than twenty thousands were open to CL, whereas only 20% of those whose income was more than twenty thousand preferred CL. The respondents’ knowledge of eye drops ranged from 20 to 25% across the different income groups, except for those with incomes of less than 5000 SR (response rate of 11%). Finally, participants’ preference for near vision correction varied based on income (Fig. 4), where the higher the respondents’ income, the more likely they were to choose spectacles over other management procedures, although this difference was not statistically significant (χ2(4) = 28.25, *p* = 0.10).

## Discussion

The prevalence of refractive error in adults (between 16 and 40 years of age) in Saudi Arabia was investigated in two recent studies [35, 36], which revealed the distribution of refractive error by age and sex. Specifically, presbyopia impacts the quality of life of people in addition to its impact on the country’s economy [37]. Consequently, addressing presbyopia is crucial to achieving sustainable advances in promoting health and well-being for all [33]. In this study, awareness and attitudes towards presbyopia and its management approaches were investigated. Importantly, this is the first study to explore such a significant age-related health concern.

In this study, only one-third of the respondents received regular eye exams, which may suggest a need to promote ocular health and well-being. Two-thirds of the respondents were not aware of presbyopia, which is consistent with previous findings [8, 34, 38, 39]. This lack of awareness could be considered a prominent reason for the 48% prevalence of uncorrected presbyopia observed in this study. The prevalence of uncorrected presbyopia in several parts of the world ranges from 28 to 63% among adults aged > 30 years [40, 41]. Uncorrected presbyopia remains a challenging issue for patients over 40 years of age as well as eye care professionals [42]. The majority of the respondents accepted spectacles as a management method and did not suffer from social stigma while using them. Thus, promotion of presbyopia correction via spectacles could be an effective way to address the challenges faced at near distances.

The respondents overwhelmingly were not aware of management choices other than spectacles. However, when offered other options, 40% and 66% of them agreed with the use of CL and eye drops, respectively. A good proportion (10%) thought that they did not need correction. These findings highlight the necessity of educating patients about presbyopia, its management choices, side effects, and consequences. For example, issues related to the use of CL, including cost, a lack of suitable near vision correction, and an increase in dry eye with age, should be discussed with potential candidates [43, 44].

Not surprisingly, respondents’ age, sex, education, and income were found to determine their needs and preferences. For example, as patients aged, they were more likely to require near vision correction. Therefore, older patients should have easy access to eye care services. Furthermore, younger participants tended to be more open to using eye drops for presbyopia management (when approved by the Saudi FDA); this finding should encourage optometrists to open discussions with patients in younger age groups. Gender also played a significant role, with women being more aware of presbyopia and multifocal CL and preferring the use of CL and eye drops. Therefore, eye care practitioners should consider these findings when managing their patients. Additionally, residents in different local areas had different preferences, the basis behind these discrepancies is not clear yet and might be due to psychosocial factors and future studies would be required to investigate this issue. This finding also could emphasize the need to discuss all management choices with every patient to understand their preferences. It has also been observed that participants outside the central region depend heavily on optical shops and private medical complexes rather than government hospitals and primary health centres; it should be noted that the patients’ visit at the government hospitals and primary health centres will be free of charge while in the private practices will be self-paid by the patients. This finding may encourage policy makers to design routine eye-health check-up programmes for those residence areas in government hospitals or provide healthcare vouchers to lower the financial burden on those patients living outside the central region. Education was the third factor impacting responses; therefore, people with fewer education certificates may need more attention to increase awareness of presbyopia, its management choices, side effects and consequences. Interestingly, people with higher incomes were more conservative in their management choices, potentially because they were more lenient toward the use of spectacles, which is an obvious practical choice. This finding contradicts the expected assumption that people with higher incomes might be more open to other, more expensive management choices, such as eye drops and refractive surgery. Therefore, practitioners should avoid assumptions and instead discuss all options with every patient. This suggestion is also supported by previous studies, which concluded that information about presbyopia and its management approaches should be provided by eye care practitioners [38, 45]. Finally, it has been suggested that comfort and convenience are much more important than cost, given that the intervention was visually comfortable and was consistent with the patient’s lifestyle [38].

## Conclusion

Presbyopia is an ocular disorder that can be easily corrected using spectacles. The findings of this study stress the need for health education on presbyopia among the older population. Eye care practitioners should match the need to examine and correct presbyopia within the population in their mid-thirties as well as the older population. Health policy makers should include the detection and management of presbyopia as a part of any national eye care programmes.

## Data Availability

All relevant data have been provided in the manuscript. The supplementary datasets used and/or analysed during the current study are available from the corresponding author upon reasonable request.

## References

[1] Katz JA, Karpecki PM, Dorca A, Chiva-Razavi S, Floyd H, Barnes E, Wuttke M, Donnenfeld E. Presbyopia - A Review of Current Treatment Options and Emerging Therapies. Clin Ophthalmol. 2021;15:2167–78. 34079215 10.2147/OPTH.S259011 PMC8163965

[2] Glasser A, Campbell MCW. Presbyopia and the optical changes in the human crystalline lens with age. Vision Res. 1998;38(2):209–29. 9536350 10.1016/S0042-6989(97)00102-8

[3] Goertz AD, Stewart WC, Burns WR, Stewart JA, Nelson LA. Review of the impact of presbyopia on quality of life in the developing and developed world. Acta Ophthalmol. 2014;92(6):497–500. 24910300 10.1111/aos.12308

[4] World Report on Vision. efaidnbmnnnibpcajpcglclefindmkaj/https://www.who.int/docs/default-source/documents/publications/world-vision-report-accessible.pdf.

[5] Fricke TR, Tahhan N, Resnikoff S, Papas E, Burnett A, Ho SM, Naduvilath T, Naidoo KS. Global Prevalence of Presbyopia and Vision Impairment from Uncorrected Presbyopia: Systematic Review, Meta-analysis, and Modelling. Ophthalmology. 2018;125(10):1492–9. 29753495 10.1016/j.ophtha.2018.04.013

[6] Wolffsohn JS, Davies LN. Presbyopia: Effectiveness of correction strategies. Prog Retin Eye Res. 2019;68:124–43. 30244049 10.1016/j.preteyeres.2018.09.004

[7] Frick KD, Joy SM, Wilson DA, Naidoo KS, Holden BA. The Global Burden of Potential Productivity Loss from Uncorrected Presbyopia. Ophthalmology. 2015;122(8):1706–10. 26190438 10.1016/j.ophtha.2015.04.014

[8] Chan VF, MacKenzie GE, Kassalow J, Gudwin E, Congdon N. Impact of Presbyopia and Its Correction in Low- and Middle-Income Countries. Asia Pac J Ophthalmol. 2018;7(6):370–4. 10.22608/APO.2018449 30523677

[9] Holden BA, Fricke TR, Ho SM, Wong R, Schlenther G, Cronjé S, Burnett A, Papas E, Naidoo KS, Frick KD. Global Vision Impairment Due to Uncorrected Presbyopia. Arch Ophthalmol. 2008;126(12):1731–9. 19064856 10.1001/archopht.126.12.1731

[10] He M, Abdou A, Ellwein LB, Naidoo KS, Sapkota YD, Thulasiraj RD, Varma R, Zhao J, Kocur I, Congdon NG. Age-related prevalence and met need for correctable and uncorrectable near vision impairment in a multi-country study. Ophthalmology. 2014;121(1):417–22. 23993359 10.1016/j.ophtha.2013.06.051 PMC6029939

[11] Sheppard AL, Wolffsohn JS. Digital eye strain: prevalence, measurement and amelioration. BMJ Open Ophthalmol. 2018;3(1):2018–000146. 10.1136/bmjophth-2018-000146 PMC6020759 29963645

[12] Coles-Brennan C, Sulley A, Young G. Management of digital eye strain. Clin Exp Optom. 2019;102(1):18–29. 29797453 10.1111/cxo.12798

[13] McDonnell PJ, Lee P, Spritzer K, Lindblad AS, Hays RD. Associations of presbyopia with vision-targeted health-related quality of life. Arch Ophthalmol. 2003;121(11):1577–81. 14609914 10.1001/archopht.121.11.1577

[14] Lu Q, Congdon N, He X, Murthy GV, Yang A, He W. Quality of life and near vision impairment due to functional presbyopia among rural Chinese adults. Invest Ophthalmol Vis Sci. 2011;52(7):4118–23. 21508106 10.1167/iovs.10-6353

[15] Wolffsohn JS, Leteneux-Pantais C, Chiva-Razavi S, Bentley S, Johnson C, Findley A, Tolley C, Arbuckle R, Kommineni J, Tyagi N. Social Media Listening to Understand the Lived Experience of Presbyopia: Systematic Search and Content Analysis Study. J Med Internet Res. 2020;22(9):18306. 10.2196/18306 PMC7536603 32955443

[16] Priyambada S. Premature Presbyopia and its Risk Factors - A Hospital based Study. Int J Contemp Med Res. 2019;6(3):C1–4.

[17] McDonald MB, Barnett M, Gaddie IB, Karpecki P, Mah F, Nichols KK, Trattler WB. Classification of Presbyopia by Severity. Ophthalmol Ther. 2022;11(1):1–11. 34709607 10.1007/s40123-021-00410-w PMC8770716

[18] Lord SR, Dayhew J, Howland A. Multifocal glasses impair edge-contrast sensitivity and depth perception and increase the risk of falls in older people. J Am Geriatr Soc. 2002;50(11):1760–6. 12410892 10.1046/j.1532-5415.2002.50502.x

[19] Johnson L, Buckley JG, Scally AJ, Elliott DB. Multifocal spectacles increase variability in toe clearance and risk of tripping in the elderly. Invest Ophthalmol Vis Sci. 2007;48(4):1466–71. 17389472 10.1167/iovs.06-0586

[20] Evans BJ. Monovision: a review. Ophthalmic Physiol Opt. 2007;27(5):417–39. 17718882 10.1111/j.1475-1313.2007.00488.x

[21] Gispets J, Arjona M, Pujol J, Vilaseca M, Cardona G. Task oriented visual satisfaction and wearing success with two different simultaneous vision multifocal soft contact lenses. Journal of Optometry. 2011;4(3):76–84. 10.1016/S1888-4296(11)70046-2

[22] Gil-Cazorla R, Shah S, Naroo SA. A review of the surgical options for the correction of presbyopia. Br J Ophthalmol. 2016;100(1):62–70. 25908836 10.1136/bjophthalmol-2015-306663

[23] Seyeddain O, Bachernegg A, Riha W, Rückl T, Reitsamer H, Grabner G, Dexl AK. Femtosecond laser-assisted small-aperture corneal inlay implantation for corneal compensation of presbyopia: two-year follow-up. J Cataract Refract Surg. 2013;39(2):234–41. 23245507 10.1016/j.jcrs.2012.09.018

[24] Seyeddain O, Hohensinn M, Riha W, Nix G, Rückl T, Grabner G, Dexl AK. Small-aperture corneal inlay for the correction of presbyopia: 3-year follow-up. J Cataract Refract Surg. 2012;38(1):35–45. 22018596 10.1016/j.jcrs.2011.07.027

[25] Pallikaris IG, Panagopoulou SI. PresbyLASIK approach for the correction of presbyopia. Curr Opin Ophthalmol. 2015;26(4):265–72. 26058023 10.1097/ICU.0000000000000162

[26] Ambrósio R Jr. Post-LASIK Ectasia: Twenty Years of a Conundrum. Semin Ophthalmol. 2019;34(2):66–8. 30664391 10.1080/08820538.2019.1569075

[27] Benozzi G, Perez C, Leiro J, Facal S, Orman B. Presbyopia Treatment With Eye Drops: An Eight Year Retrospective Study. Transl Vis Sci Technol. 2020;9(7):25. 32832231 10.1167/tvst.9.7.25 PMC7414614

[28] Abdelkader A. Improved Presbyopic Vision With Miotics. Eye Contact Lens. 2015;41(5):323–7. 25806674 10.1097/ICL.0000000000000137

[29] Elliott W, Chan J. Pilocarpine Hydrochloride Ophthalmic Solution 1.25% (Vuity). Inter Med. 2021;43(24).

[30] Price FW Jr, Hom M, Moshirfar M, Evans D, Liu H, Penzner J, Robinson MR, Lee S, Wirta DL. Combinations of Pilocarpine and Oxymetazoline for the Pharmacological Treatment of Presbyopia: Two Randomized Phase 2 Studies. Ophthalmol Sci. 2021;1(4):100065. 36246939 10.1016/j.xops.2021.100065 PMC9562347

[31] Waring GOt, Brujic M, McGee S, Micheletti JM, Zhao C, Schachter S, Liu H, Safyan E: Impact of presbyopia treatment pilocarpine hydrochloride 1.25% on night-driving performance. Clin Exp Optom 2023:1–8. 10.1080/08164622.2023.2279189 38044272

[32] Mercer RN, Milliken CM. Waring GOt, Rocha KM: Future Trends in Presbyopia Correction. J Refract Surg. 2021;37(S1):S28–34. 34170762 10.3928/1081597X-20210408-06

[33] Yang A, Lim SY, Wong YL, Yeo A, Rajeev N, Drobe B. Quality of Life in Presbyopes with Low and High Myopia Using Single-Vision and Progressive-Lens Correction. J Clin Med. 2021;10(8):1589. 33918687 10.3390/jcm10081589 PMC8069619

[34] Gajapati CV, Pradeep AV, Kakhandaki A, Praveenchandra RK, Rao S. Awareness of Presbyopia among Rural Female Population in North Karnataka. J Clin Diagn Res. 2017;11(9):1. 10.7860/JCDR/2017/26125.10608 PMC5713766 29207744

[35] Almudhaiyan T, Alhamzah A, AlShareef M, Alrasheed A, Jaffar R, Alluhidan A, Al-Hazazi M, Aldebasi T. The prevalence of refractive errors among Saudi adults in Riyadh. Saudi Arabia Saudi J Ophthalmol. 2020;34(1):30–4. 33542984 10.4103/1319-4534.301297 PMC7849861

[36] Parrey MUR, Elmorsy E. Prevalence and pattern of refractive errors among Saudi adults. Pak J Med Sci. 2019;35(2):394–8. 31086521 10.12669/pjms.35.2.648 PMC6500803

[37] Berdahl J, Bala C, Dhariwal M, Lemp-Hull J, Thakker D, Jawla S. Patient and Economic Burden of Presbyopia: A Systematic Literature Review. Clin Ophthalmol. 2020;14:3439–50. 33116396 10.2147/OPTH.S269597 PMC7588278

[38] Hutchins B, Huntjens B. Patients’ attitudes and beliefs to presbyopia and its correction. J Optometry. 2021;14(2):127–32. 10.1016/j.optom.2020.02.001 PMC8093526 32241701

[39] Weale RA. Epidemiology of refractive errors and presbyopia. Surv Ophthalmol. 2003;48(5):515–43. 14499819 10.1016/S0039-6257(03)00086-9

[40] He M, Abdou A, Naidoo KS, Sapkota YD, Thulasiraj RD, Varma R, Zhao J, Ellwein LB. Prevalence and correction of near vision impairment at seven sites in China, India, Nepal, Niger, South Africa, and the United States. Am J Ophthalmol. 2012;154(1):107–16. 22534109 10.1016/j.ajo.2012.01.026

[41] Patel I, Munoz B, Burke AG, Kayongoya A, McHiwa W, Schwarzwalder AW, West SK. Impact of presbyopia on quality of life in a rural African setting. Ophthalmology. 2006;113(5):728–34. 16650665 10.1016/j.ophtha.2006.01.028

[42] Ong HS, Evans JR, Allan BD. Accommodative intraocular lens versus standard monofocal intraocular lens implantation in cataract surgery. Cochrane Database Syst Rev. 2014;1(5):CD009667. 10.1002/14651858.CD009667.pub2 PMC10505746 24788900

[43] Begley CG, Caffery B, Nichols KK, Chalmers R. Responses of contact lens wearers to a dry eye survey. Optom Vis Sci. 2000;77(1):40–6. 10654857 10.1097/00006324-200001000-00012

[44] Dumbleton K, Woods CA, Jones LW, Fonn D. The impact of contemporary contact lenses on contact lens discontinuation. Eye Contact Lens. 2013;39(1):93–9. 23266586 10.1097/ICL.0b013e318271caf4

[45] Fylan F, Grunfeld EA. Visual illusions? Beliefs and behaviours of presbyope clients in optometric practice. Patient Educ Couns. 2005;56(3):291–5. 15721971 10.1016/j.pec.2004.03.003

